# A Novel and Efficient Gene Transfer Strategy Reduces Glial Reactivity and Improves Neuronal Survival and Axonal Growth *In Vitro*

**DOI:** 10.1371/journal.pone.0006227

**Published:** 2009-07-14

**Authors:** Mathieu Desclaux, Marisa Teigell, Lahouari Amar, Roland Vogel, Minerva Gimenez y Ribotta, Alain Privat, Jacques Mallet

**Affiliations:** 1 Biotechnology and Biotherapy, Centre de Recherche de l'Institut du Cerveau et de la Moelle Epiniere, Centre National de la Recherche Scientifique (CNRS) UMR 7225, Institut National de la Santé et de la Recherche Médicale (INSERM) UMRS 975, Université Pierre et Marie Curie (UPMC) - Hôpital de la Pitié Salpêtrière, Paris, France; 2 NEUREVA-inc., Montpellier, France; 3 Consejo Superior de Investigationes Cientifícas (CSIC), Universidad Miguel Hernández (UMH), Instituto de Neurociencias de Alicante, Sant Joan D'Alacant Nacional, España; 4 Institut National de la Santé et de la Recherche Médicale (INSERM) U583, Physiopathologie et Thérapie des Déficits Sensoriels et Moteurs, Institut des Neurosciences de Montpellier (INM), Université Montpellier 2, Montpellier, France; University of North Dakota, United States of America

## Abstract

**Background:**

The lack of axonal regeneration in the central nervous system is attributed among other factors to the formation of a glial scar. This cellular structure is mainly composed of reactive astrocytes that overexpress two intermediate filament proteins, the glial fibrillary acidic protein (GFAP) and vimentin. Indeed, *in vitro*, astrocytes lacking GFAP or both GFAP and vimentin were shown to be the substrate for increased neuronal plasticity. Moreover, double knockout mice lacking both GFAP and vimentin presented lower levels of glial reactivity *in vivo*, significant axonal regrowth and improved functional recovery in comparison with wild-type mice after spinal cord hemisection. From these results, our objective was to develop a novel therapeutic strategy for axonal regeneration, based on the targeted suppression of astroglial reactivity and scarring by lentiviral-mediated RNA-interference (RNAi).

**Methods and Findings:**

In this study, we constructed two lentiviral vectors, Lv-shGFAP and Lv-shVIM, which allow efficient and stable RNAi-mediated silencing of endogenous GFAP or vimentin *in vitro*. In cultured cortical and spinal reactive astrocytes, the use of these vectors resulted in a specific, stable and highly significant decrease in the corresponding protein levels. In a second model — scratched primary cultured astrocytes — Lv-shGFAP, alone or associated with Lv-shVIM, decreased astrocytic reactivity and glial scarring. Finally, in a heterotopic coculture model, cortical neurons displayed higher survival rates and increased neurite growth when cultured with astrocytes in which GFAP and vimentin had been invalidated by lentiviral-mediated RNAi.

**Conclusions:**

Lentiviral-mediated knockdown of GFAP and vimentin in astrocytes show that GFAP is a key target for modulating reactive gliosis and monitoring neuron/glia interactions. Thus, manipulation of reactive astrocytes with the Lv-shGFAP vector constitutes a promising therapeutic strategy for increasing glial permissiveness and permitting axonal regeneration after central nervous system lesions.

## Introduction

Astrocytes are essential companions of neurons from early stage of the central nervous system (CNS) formation. As radial glial cells, they support neuronal migration [1]. At adult stages, they have major functions in neuronal homeostasis [2], [3], formation and maintenance of the blood-brain barrier [4], control of synaptic communication or immune response modulation [5], [6], [7], [8]. In the injured CNS, reactive astrocytes undergo important morphological modifications, such as hyperplasia and hypertrophy [9], [10], and express a broad range of molecules with growth inhibitory properties, such as chondroitin sulfate proteoglycans and tenascin-C [11], [12], [13], [14], [15]. Thus reactive astrocytes among other cell types such as microglial cells, oligodendrocytes precursors, Schwann cells or fibroblasts, are mainly involved in the formation of both a physical and a biochemical barrier to axonal regeneration: the glial scar [10], [16].

The main molecular hallmark of reactive astrocytes is the upregulation of two intermediate filament (IF) proteins, the glial fibrillary acidic protein (GFAP) and vimentin, which are the major components of astrocytes cytoskeleton [17], [18], [19]. The expression of these two proteins is tightly regulated during the development and is associated with the astrocytic differentiation [20], [21]. At adult stages the exact physiological roles of the GFAP and vimentin in the astrocytes remain incompletely understood, but they appear to be involved in the maintenance of shape, mechanical stability, or in the vesicle mobility in astrocytes [22], [23], [24]. Physiologically, GFAP is also necessary for the integrity of the blood-brain barrier [25], [26], the maintenance of CNS cytoarchitecture [25], [27], and of synaptic function [28]. After CNS injury, the upregulation of both GFAP and vimentin expression during astrogliosis play a major role in astrocytic motility [29] and hypertrophy [17], [30], which are key properties of reactive astrocytes contributing to the glial scar [16]. It has been therefore suggested that the overproduction of GFAP and vimentin after CNS lesions could be used as a specific target for neuronal repair strategies.

A series of experiments with mutant mice lacking GFAP and vimentin have demonstrated the consequences of the absence of both proteins on astrocytes reactivity and axonal plasticity *in vitro* and *in vivo*. Survival of cortical neurons and neurite outgrowth was improved in cocultures with GFAP −/− or GFAP −/− Vim −/− spinal astrocytes [31]. *In vivo*, double knockout mice lacking GFAP and vimentin presented lower than normal levels of astroglial activity, with a reduced astrocytic hypertrophy and a defective scar formation [30], [32], [33]. Moreover GFAP −/− Vim −/− mice showed significant axonal regrowth of the descending fibers of the corticospinal tract (CST) and ventral horn serotonergic tract, associated with an improved functional recovery after spinal cord hemisection [32]. These mice also presented improved axonal regeneration after lesion of entorhinal cortex [30]. However, no CNS repair strategies based solely on the manipulation of the astrogliosis have yet been developed. The modulation of astrogliosis through posttranscriptional inhibition of GFAP and vimentin production may provide a promising new strategy with potential clinical applications for promoting axonal regeneration after formation of a CNS lesion.

Long-term, stable inhibition of the expression of single genes has been successfully achieved in mammalian cells, using lentiviral vectors mediating the constitutive expression of short-hairpin RNAs (shRNAs), which are rapidly cleaved into small-interfering RNAs (siRNAs) *in situ* [34], [35]. Thus, RNA-interference (RNAi) appears to be a remarkable tool with potential applications for use in clinical practice, for the treatment of diseases caused by a defective gene, which needs to be inhibited. In this study, we aimed at silencing the expression of the GFAP and vimentin genes through a lentiviral-mediated RNAi approach, in order to reduce astrogliosis in reactive astrocytes. We first identified target sequences in the GFAP and vimentin mRNAs that might enable us to decrease efficiently the levels of both proteins. We then produced lentiviral vectors allowing constitutive expression of the corresponding shRNA sequences. We show that these vectors induced a strong, stable decrease in endogenous GFAP and vimentin expression in primary cultured astrocytes. These vectors also improve neuronal survival and neurite outgrowth and prevent glial scarring in an *in vitro* model using scratched astrocytes.

## Materials and Methods

### Ethical Statements

All animals were handled in strict accordance with good animal practice as defined by the French animal welfare bodies, and all animal work was approved by the Direction Départmentale des Services Vétérinaires de Paris and by the Direction Départementale des Services Vétérinaires de l'Hérault.

### Plasmid construction

All the primers used in this study were synthesized by Eurogentech, (Angers, France) and all the restriction enzymes were produced by New England Biolabs (Ipswich, MA). We first constructed plasmids encoding mouse GFAP and/or mouse vimentin fused to the Enhanced Green Fluorescent Protein (EGFP). The murine GFAP and vimentin cDNA sequences were obtained by RT-PCR, using the following primers: 5′-GGGAATTCATGCCTCCGAGACGGTGGTC-3′ (GFAP sense strand), 5′- GCGATCCATCACCACGTCCTTGTGCTC-3′ (GFAP antisense strand), 5′-GCGAATTCCAAGCCAGCCCACCTTCGA-3′ (vimentin sense strand) and 5′-GCGGATCCAGTTCAAGGTCATCGTGATGCTG-3′ (vimentin antisense strand). The murine GFAP and vimentin cDNAs were then inserted between the *Eco*RI and *Bam*HI restriction sites in pEGFP-N1 (Clontech, Mountain View, CA).

We designed 66-nucleotide potential shRNA sequences to silence the expression of the mouse GFAP or vimentin genes in a specific manner as previously described (Rubinson 2003). Each shRNA sequence comprises a selected siRNA sequence targeting the murine GFAP mRNA (NCBI Accession Number NM_010277) or murine vimentin mRNA (NCBI Acession Number NM_011701) in both sense and antisense orientations, separated by a 9 nucleotides loop (5′-TTCAAGAGA-3′) and followed by five thymidine residues as an RNA polymerase III transcription termination signal. *Bbs*I and *Bam*HI restriction sites were incorporated at the 5′ and 3′ ends, respectively. Potential siRNA sequences were selected empirically, as described by Tuschl *et al*., and with the help of the Qiagen siRNA design tool (Qiagen, Valencia, CA; http://www.biopredsi.org/design.html) and the Whitehead Institute for Biomedical Research (http://jura.wi.mit.edu/bioc/siRNAext/), [36]. The DNA fragment encoding each shRNA was generated by annealing two complementary oligonucleotides (Eurogentech) and the resulting double-stranded DNA fragments were inserted between the *Bbs*I and *Bam*HI sites of pcDNA-ΔU6wt. We obtained pcDNA-ΔU6wt from pcDNA3.0, by replacing the original CMV promoter with the U6 promoter, which was obtained by RT-PCR from HEK 293-T cells.

For the production of lentiviral vectors, cassettes allowing effective shGFAP or shVIM expression under control of the U6 promoter were recovered from the pcDNA-ΔU6wt-shRNA and inserted into the lentivector precursor plasmid pFlap-PGK-EGFP-WPRE. This plasmid is a derivative of pTrip-CMVmin-WPRE, as described by Vogel *et al.* [37], from which the CMVmin element has been deleted [38]. This element was removed by *Bam*HI-*Kpn*I digestion and replaced by a multicloning site (MCS) containing single restriction sites for *Sal*I and *Nhe*I. The PGK sequence was amplified by PCR from pTrip-PGK-EGFP, with primers incorporating a *Bam*HI restriction site 5′ to the PGK gene (5′-CGGGATCCTGCTCGAGTATTCTACCGGGTAGGGGAGGCG-3′) and a *Sal*I restriction site 3′ to the gene (5′-GCTGGGTCGACTCGAAAGGCCCGGAGATGAGG-3′). The EGFP coding sequence was amplified by PCR from pEGFP-N1 (Clontech), with primers incorporating a *Sal*I restriction site 5′ to the gene (5′-GTGGGTCGACCTCGCCACCATGGTGAGCAAGGGC-3′) and an *Xba*I restriction site 3′ to the gene (5′-GCTCTAGAAGCTAGAGTCCGGCCGCTTTACTTGTAC-3′). The WPRE sequence was amplified by PCR from pTrip-U6min-shGFP-WPRE, with primers incorporating an *Xba*I restriction site 5′ to the gene (5′-GCGGATCCATGTCGACGTAGCTAGCGATAATCAACCTCTGGATTACAAAATTTGTG-3′) and a *Kpn*I restriction site 3′ to the gene (5′-GGGGTACCATCCGATGCGGGGAGGCGGC-3′). The various PCR products were sequentially subcloned into pTrip-U6min-shGFP-MCS, using the appropriate restriction sites. Finally, the U6-shGFAP or U6-shVIM cassette was inserted into pFlap-PGK-EGFP-WPRE between the *Mlu*I and *Bam*HI restriction sites.

As controls, a lentiviral vector expressing only EGFP under control of the PGK promoter, a lentiviral vector producing a “scrambled” shRNA sequence, and a lentiviral vector producing a shRNA directed against the OB-RGRP transcript and previously described (Couturier *et al.* 2007) were produced.

### HEK-293T cell line culture, transfection and lentivirus production

#### HEK 293 T cell culture

The HEK-293T cell line (ATCC # CRL-11268) was cultured at 37°C, under a humidified 5% CO_2_/95% air atmosphere, in Dulbecco's modified Eagle medium (DMEM, Invitrogen, Carlsbad, CA) supplemented with 10% fetal bovine serum (FBS, Invitrogen) and 1% antibiotic solution (mixture of 10 U/ml penicillin G and 10 µg/ml streptomycin, Eurobio, Courtaboeuf, France).

#### Transient transfection of HEK 293 T cells for shRNA screening

HEK 293 T cells were plated in 40 mm dishes and transfected with a total of 4 µg of DNA per dish, using the calcium phosphate method. For each transfection, the molar ratio of plasmid encoding fusion protein construct/plasmids encoding shRNAs was 1∶20. Culture medium was removed six hours after transfection and replaced with fresh medium. Cells were fixed in 4% paraformaldehyde (PFA) in PBS, 72 hours after cotransfection and EGFP levels were analyzed with a FACScan flow cytometer (Becton Dickinson, Franklin Lake, NJ). Results were given as mean percentages±SEM. Statistical comparisons were done using paired T-test. Significance was accepted at p<0.05.

#### Lentiviral vector production

Lentiviral vector particles were produced by the calcium phosphate cotransfection method, as previously described [39], [40]. Cells were cotransfected with the vector plasmid, an encapsidation plasmid (p8.9) [41], and a plasmid encoding the vesicular stomatitis virus envelope glycoprotein (pMD-G), in a culture medium containing chloroquine (2,5 µM, Sigma-Aldrich). The medium was replaced 6 hours after transfection and collected 36 hours later. Supernatants were treated with DNAse I (Roche) and subjected to ultracentrifugation at 22 000 rpm (rotor SW28, Beckman-Coulter) for 90 minutes. The resulting pellet was resuspended in 0.1 M PBS, aliquoted and frozen at −80°C until use.

The HIV p24 gag antigen was quantified for each stock by ELISA (HIV-1 P24 antigen assay, Beckman Coulter, Fullerton, CA). EGFP-expressing vectors were titrated by transducing 100 000 HEK 293 T cells in 24-well plates with a serial dilution (4 µl, 4×10^−1^ µl, 4×10^−2^ µl, 4×10^−3^ µl, 4×10^−4^ µl, 4×10^−5^ µl). Cells were harvested by centrifugation and resuspended in a fixative solution of 1% PFA, 72 hours after transduction. The number of EGFP-positive cells was determined in a FACScan flow cytometer (Beckton Dickinson).

### Primary glial cell line cultures and lentiviral transductions

#### Glial cell culture

Primary cultures of glial cells were established from the spinal cord (SC) or cortices of two-day-old C57/Bl6 mice (Janvier, Le Genest-St-Isle, France). The animals were sacrificed in aseptic conditions. The SC and cerebral cortices were dissected, freed from meninges and collected in cold HBSS supplemented with calcium and magnesium (Invitrogen), glucose (6 g/L) and 1% antibiotic solution (Invitrogen). The fragments of SC and cortex were then treated with 0.25% trypsin-EDTA (Eurobio) and DNAse A (50 µg/ml, Roche, Neuilly-sur-Seine, France) for 8 minutes at 37°C. Trypsin was then inactivated by adding 10% FBS. Tissues were rinsed in HBSS without calcium and magnesium (Invitrogen) and resuspended in 1 ml of culture medium consisting of a 1∶1 mixture of DMEM and Ham's F12 medium (DMEM/F12 1∶1, Invitrogen) supplemented with 10% FBS, glucose (6 g/L) and antibiotics. Cells were mechanically dissociated, resuspended in the same culture medium and plated at a final concentration of ∼200 000 cells/well on glass coverslips treated with 50 µg/ml of low-molecular weight poly-D-lysine (Sigma-Aldrich, St Louis, MO) in 24-well dishes (Nunc, Roskilde, Danemark), for immunocytochemical analyses. For immunoblotting studies, cells were plated in six-well dishes (TPP, Trasadingen, Swizerland), with one cortical hemisphere per well, or one spinal cord per well. The culture medium was completely replaced by fresh cold medium after 24 hours and weekly thereafter.

#### In vitro transduction of glial cells

One week after seeding, glial cells were transduced with the lentiviral vectors. The medium was removed and replaced with lentiviral vector diluted in DMEM/F12 medium without FBS but with antibiotics. Cells were incubated overnight with this lentiviral vector-medium mixture (500 µl/well for 24-well plates and 1 ml/well for 6-well plates). After 24 hours, the infection medium was removed and replaced with fresh DMEM/F12 medium supplemented with FBS. The amount of lentiviral vector applied to the cells is expressed as a multiplicity of infection (MOI), corresponding to the number of transducing particles (TU) per cell.

### Glial cell scratch wound assay

We used two different paradigms for the scratch wound assay. In the first paradigm, confluent glial cell monolayers were scratched with a sterile 20 to 200 µm plastic pipette tip (Corning, NY) two weeks after lentiviral transduction. Cells were then washed twice with PBS and the medium was replaced with fresh DMEM/F12 medium supplemented with glucose, FBS and antibiotics. Cells were fixed with 4% paraformaldehyde (PFA, Sigma-Aldrich) in PBS after 48 hours, one week and two weeks of incubation.

In the second paradigm, confluent glial cell monolayers were first scratched as described above, and the cells were then immediately transduced with the lentiviral vectors. The medium was removed 24 hours after transduction and the cells were fixed in 4% PFA in PBS 48 hours, one week and two weeks after scratching.

### Western blot analysis

Proteins were extracted from glial cell cultures two weeks after transduction, for western blotting. Glial cells were washed in PBS, scraped from the dishes or plates into PBS and centrifuged at 1000 g for 10 minutes. The cell pellets were washed again, resuspended in PBS and centrifuged at 4°C and at 10 000 g. Proteins were extracted from the cell pellets in lysis buffer, as previously described [42]. Protein concentration was determined by the Bradford method. Equal loads of proteins (20 µg) were subjected to electrophoresis in 9% polyacrylamide gels in the presence of SDS and the resulting bands were blotted onto a nitrocellulose membrane in transfer buffer at 50 mA overnight. The membrane was blocked by incubation with 5% skimmed milk in PBS-Tween 0.1% for 1 hour, and was then incubated with the primary antibody — (1) rabbit polyclonal IgG directed against GFAP (1∶100 000, Dako, Glostrup, Denmark) or (2) mouse monoclonal IgG directed against vimentin (RV202 clone, 1∶1000, BD-Pharmingen) — overnight at 4°C or for two hours at room temperature. The membrane was then incubated with secondary antibody: peroxidase-conjugated anti-rabbit (1∶5000) or anti-mouse (1∶5000) IgG (Amersham). The binding of antibodies to proteins was detected by enhanced chemiluminescence, using an ECL detection kit for western blots (Amersham).

### Immunocytochemical analysis of glial cell monocultures

Two or three weeks after transduction, the cells were fixed by incubation in 4% PFA in PBS for 30 minutes to one hour at room temperature. The cells were then washed twice in PBS and processed for immunostaining. Cells were first incubated for one hour with a saturated 2% bovine serum albumin solution (BSA, Sigma-Aldrich) and 0.1% Triton (Triton X-100, Sigma-Aldrich) in PBS. They were then incubated overnight with the following primary antibodies: (1) rabbit polyclonal IgG directed against GFAP (1∶10 000, Dako), (2) mouse monoclonal IgG directed against GFP (AbCam, 1∶500) and (3) mouse monoclonal IgM directed against vimentin (clone IL6, 1∶500, Sigma-Aldrich). All primary antibodies were diluted in PBS supplemented with 2% BSA and 0.1% Triton. Following incubation for 24 h at 4°C, the cultures were rinsed with PBS and incubated for 1 hour at room temperature with the following secondary antibodies: goat anti-rabbit Alexa 488 (1∶1000, Invitrogen), goat anti-mouse IgG Alexa 555 (1∶1000, Invitrogen) and goat anti-mouse IgM Cy5 (Jackson Immunoresearch Laboratories, Westgrove, PA). Cells were rinsed again with PBS and incubated with Hoechst 33342 (Sigma-Aldrich) solution for 5 minutes. Finally, the glass coverslips on which the cells were growing were briefly rinsed in water and mounted in Fluorescent Mounting Medium (Dako).

### Analysis of neuron-glial cell cocultures, neuronal survival and neurite outgrowth

#### Neuron-glial cell cocultures

Embryonic cortical neurons were cocultured directly with transduced glial cell monolayers. A neuronal suspension was prepared from the neocortex of 14-day-old C57Bl/6 mouse embryos (Janvier). The meninges were removed and the cortices were dissected in cold HBSS containing calcium and magnesium (Gibco). The tissue was then incubated in HBSS without calcium and magnesium for 5 minutes at 37°C and then at 25°C. The cells were mechanically dissociated in 1 ml of DMEM/F12 medium supplemented with N2-complement (Gibco) and antibiotics. The cells were seeded onto the surface of the confluent glial cell monolayers at an initial density of 25 000 cells/well in 24-well dishes.

#### Neuronal survival assessment and neurite outgrowth assay

The neuronal population was characterized by immunocytochemistry. After 7 days *in vitro* in coculture, cultures were fixed in 4% PFA in PBS. Cells were rinsed in PBS and incubated overnight at 4°C with a mouse monoclonal IgG antibody directed against the β-III-tubulin protein (1∶500, Sigma-Aldrich). Cells were then rinsed in PBS and incubated for one hour with a peroxidase-conjugated rabbit anti-mouse antibody (1∶100, Sigma-Aldrich). Immunoreactivity was detected by incubation with a substrate of peroxidase. The chromogen used for development was 0.1% 3,3′-diaminobenzidine (DAB) in the presence of H_2_O_2_. Glass coverslips were finally mounted in Depex after dehydration of the cells growing on them.

We measured two variables: neuronal survival and neurite outgrowth. Neuronal survival was evaluated by measuring neuronal density with Image J software (Cell Counting program). Neuronal density is expressed as a percentage vs. neurons growing on non transduced glial cell monolayers. Neurite outgrowth was evaluated by measuring the area of the immunoreactive β-III-tubulin occupied by the perikaria and determining the number of neurites per neuron. Areas were measured with an automatic, personalized threshold program, using Image J software. Three independent experiments were performed. In each experiment, three coverslips were used for each set of conditions, and ten fields per coverslip were randomly selected at a magnification of x10. Results were given as mean percentages±SEM. Statistical comparisons were carried out for each variable using one-way ANOVA (analysis of variance) with Bonferoni *post-hoc* analysis (GraphPad Prism 5 software). Significance was accepted at p<0.05.

## Results

### Identification of oligonucleotide sequences for the RNAi-mediated inhibition of GFAP and vimentin gene expression

The RNAi-mediated knockdown of protein expression depends on particular oligonucleotide sequences, the efficiency of which depends on several parameters and is specific for each animal species. We first identified interfering sequences that allow specific silencing of murine GFAP and vimentin. We screened the cDNAs encoding murine GFAP and vimentin to identify oligonucleotide sequences capable of efficiently abolishing the production of both proteins. We chose potential siRNA sequences with selection algorithms based on empirical criteria, as described in the materials and methods. A panel of ten 21-nucleotide candidate sequences was selected for each protein, at different positions within the coding region of the corresponding mRNA (**Table 1** and **Table 2**). BLAST analysis confirmed that none of these sequences displayed significant sequence identity to any other mRNA. As RNA interference triggered by shRNA constructs has been shown to be particularly efficient, we constructed plasmid vectors to express these interfering sequences as shRNAs under control of the human U6 promoter.

**Table 1:** Candidate target sites for RNAi in mouse GFAP transcripts.

Name	siRNA sequence	Position
GFAP-1	GAGCCCACCAAACTGGCTGAT	277–297
GFAP-2	GATCTATGAGGAGGAAGTTCG	560–580
**GFAP-3**	**GAGAGAGATTCGCACTCAATA**	**660–680**
GFAP-4	GAGAGATTCGCACTCAATACG	662–682
GFAP-5	GAGATTCGCACTCAATACGAG	664–684
GFAP-6	GCCGCCAACTGCAGGCCTTGA	812–832
GFAP-7	GCAGGAGTACCACGATCTAC	993–1012
**GFAP-8**	**GAGATCGCCACCTACAGGAAA**	**1038–1058**
GFAP-9	GATCGCCACCTACAGGAAATT	1040–1060
GFAP-10	GCGGGATGGTGAGGTCATTAA	1199–1219

**Table 2:** Candidate target sites for RNAi in mouse Vimentin transcripts.

Name	siRNA sequence	Position
VIM-1	GTGGAATCCTTGCAGGAAGAA	1150–1170
VIM-2	GAATCCTTGCAGGAAGAAATT	1153–1173
**VIM-3**	**GAATGGTACAAGTCCAAGTTT**	**1345–1365**
VIM-4	GATGGAAGAGAATTTTGCCCT	1518–1538
VIM-5	GTGAATACCAAGATCTGCTC	1622–1641
VIM-6	GAATACCAAGATCTGCTCAAT	1624–1644
VIM-7	GAGGAGAGCAGGATTTCTCTG	1699–1719
VIM-8	GAGAGCAGGATTTCTCTGCCT	1702–1722
VIM-9	GAACACTCCTGATTAAGACGG	1799–1819

**Figure 1:**
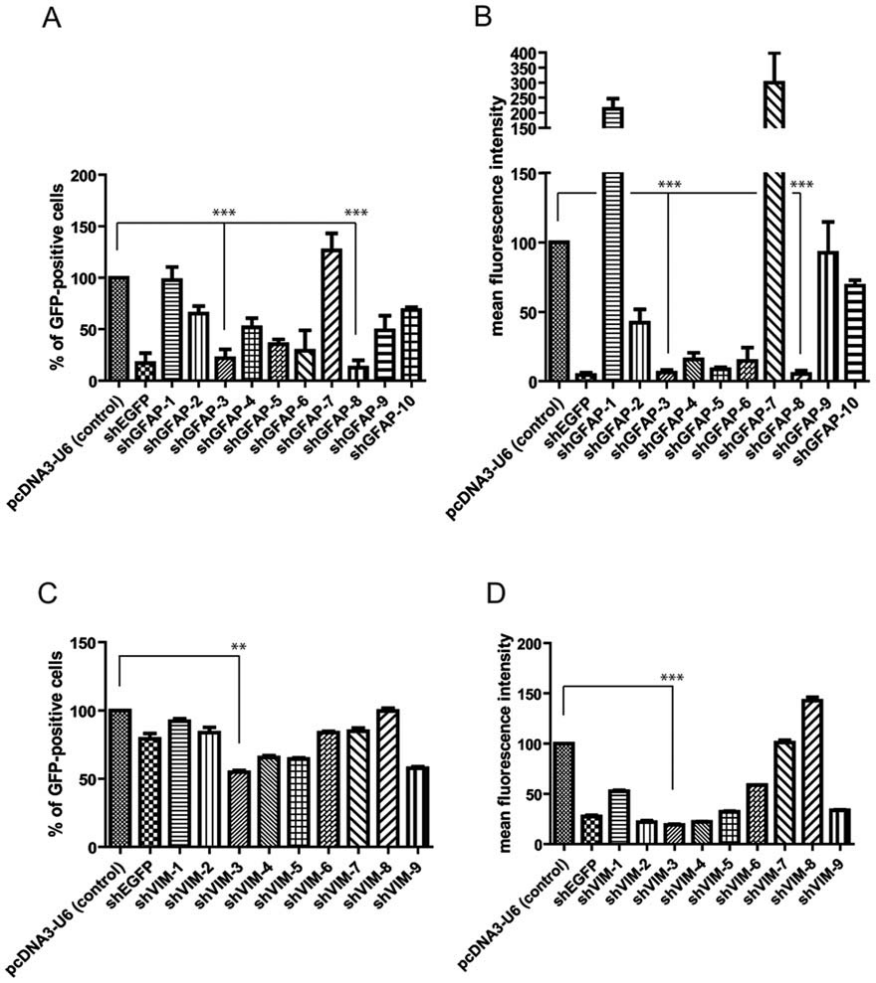
Screening of shRNAs for silencing of GFAP-EGFP and Vim-EGFP fusion protein. HEK 293 T cells were cotransfected with the GFAP-EGFP or VIM-EGFP plasmids and either pcDNA-U6wt as a control or a plasmid expressing a single shGFAP or shVIM sequence. An shRNA specific for the EGFP mRNA (pcDNA3-U6-shEGFP) was also used as a positive control. A, C: Results are expressed as the percentage of fluorescent cells, determined 72 h after transfection (**p<0.01, *** p<0.001, paired T-test). B, D: Results are expressed as mean fluorescence intensity, determined 72 h after transfection (**p<0.01, *** p<0.001, paired T-test).

We next examined the ability of the candidate sequences, shGFAP 1 to 10, and shVIM 1 to 9, to silence production of the GFAP-EGFP and Vim-EGFP fusion proteins, respectively. Each candidate shGFAP or shVIM sequence was expressed from a plasmid vector in human HEK-293 T cells cotransfected with a plasmid encoding the GFAP-EGFP or VIM-EGFP fusion protein. EGFP levels were determined by flow cytometry. We assessed two variables: the overall proportion of cells expressing EGFP and mean fluorescence intensity (MFI). Differences in the number of EGFP-expressing cells and in MFI between cells transfected with the different constructs reflect the extent to which the production of GFAP-EGFP or VIM-EGFP is inhibited and, thus, the efficiency of the GFAP- or Vim-targeting shRNAs. Two shGFAP sequences, shGFAP-3 and shGFAP-8, significantly decreased the number of GFAP-EGFP-positive cells by 83±12.81% and 87±9.4%, respectively (**Fig. 1A**). Moreover shGFAP-3 and sh-GFAP-8 also reduced mean fluorescence intensity with high significance by 96±2.62% and 96±3.11%, respectively (**Fig. 1B**). Similarly, shVIM-3 significantly decreased the number of cells positive for VIM-EGFP by 45±1.4% (**Fig. 1C**). This sequence also decreased MFI by 81±0.7% (**Fig. 1D**). Considering these results, the two sequences shGFAP-3 and shVIM-3 were selected for the production of lentiviral vectors.

### Specific lentiviral-mediated inhibition of endogenous GFAP and vimentin gene expression in medullar and cortical astrocytes

GFAP and vimentin gene expression is strong and persistent in reactive astrocytes after the occurrence of CNS lesion [43]. We assessed the cellular effects driven by the RNAi-mediated knockdown of GFAP and vimentin *in vitro*, using primary cultures of murine astrocytes, which may be considered a model of reactive astrocytes. The GFAP and vimentin genes were also strongly and persistently expressed in these cultures.

The long-term, stable silencing of GFAP and vimentin in these primary astrocyte cultures was triggered by recombinant non-replicative lentiviral vectors expressing the selected shGFAP and shvimentin sequences. These vectors, respectively called Lv-shGFAP and Lv-shVIM, also encode EGFP as a reporter protein for assessment of the transduction efficiency of the vectors. On the same molecular basis, we also constructed vectors for the expression of shRNA which inefficiently targeted GFAP or vimentin. All these lentiviral vectors were pseudotyped with the VSV envelope, which confers efficient, broad-tropism transduction *in vitro* [44].

**Figure 2:**
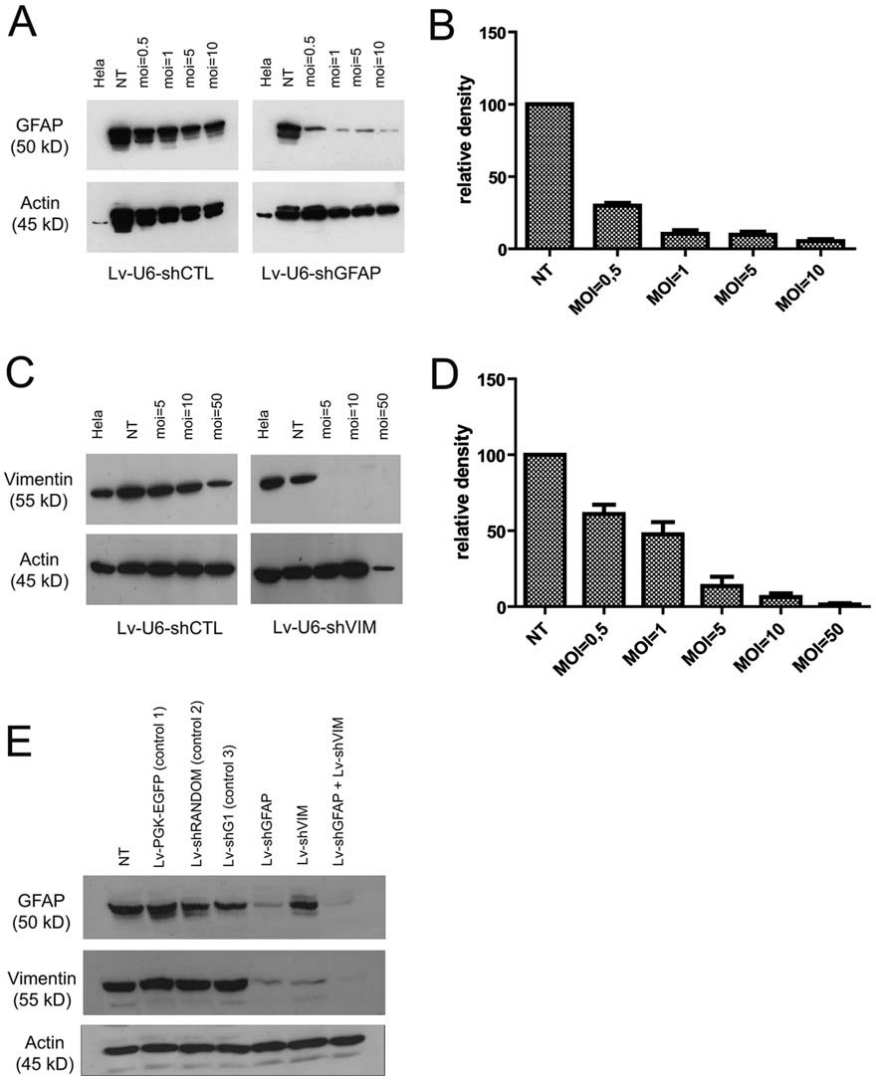
Knockdown of endogenous GFAP and vimentin gene expression in primary cultures of cortical astrocytes by lentiviral-mediated RNAi. A: GFAP levels decreased in a dose-dependent manner in cultured cortical astrocytes. Astrocytes were transduced with different amounts of the Lv-shGFAP vector one week after cell seeding. Protein levels were analyzed by western blotting 15 days after transduction with Lv-shGFAP and were compared with those for a control vector. B: Relative quantification of GFAP knockdown. At MOI = 10, GFAP levels are decreased by 90%. C: Vimentin levels decreased in a dose-dependent manner in cultured cortical astrocytes. Astrocytes were transduced with different amounts of Lv-shGFAP one week after cell seeding. Protein levels were analyzed by western blotting 15 days after transduction with the Lv-shVIM vector, and were compared with those for the control vector. D: Relative quantification of vimentin knockdown. At MOI = 10, vimentin levels are decreased by 90%. E: At MOI = 10, GFAP and vimentin levels are strongly decreased in cortical astrocytes transduced with Lv-shGFAP and Lv-shVIM, respectively. By contrast, no significant decrease in GFAP or vimentin levels was observed in astrocytes transduced with the various control vectors. Vimentin levels were also lower in astrocytes transduced with Lv-shGFAP alone. Astrocytes were transduced with the various vectors one week after cell seeding. Protein levels were analyzed by western blotting, 15 days after transduction.

The capacity of Lv-shGFAP and Lv-shVim to silence endogenous GFAP and vimentin gene expression *in vitro* was assessed by western blot analyses on cortical astrocytes. Primary cultures of cortical glial cells were transduced with various amounts of Lv-shGFAP, Lv-shVIM or the control vector. Two weeks after transduction, GFAP was detected by western blotting. Primary cultured astrocytes transduced with Lv-shGFAP vector displayed a large, dose-dependent decrease in GFAP levels with respect to the control vector (**Fig. 2A**). Western blotting showed GFAP levels to be 70% lower than those in non transfected cells, in cells transfected with 0.5 viral particles per cell (MOI = 0.5), and 95% lower in cells transfected with 10 viral particles per cell (**Fig. 2B**). Similarly, primary cultures of glial cells transduced with the Lv-shVIM vector presented a substantial, dose-dependent decrease in vimentin levels with respect to the control vector (**Fig. 2C**). Quantitative analysis also revealed that vimentin levels were decreased by more than 90% at an MOI of 10 (**Fig. 2D**).

We ascertained that the silencing of GFAP and vimentin was due to genuine specific RNAi targeting, by comparing GFAP and vimentin levels after the transduction of cortical glial cells with three additional controls: (1) a lentiviral vector encoding only EGFP under control of the PGK promoter (Lv-PGK-EGFP), (2) a vector carrying a “scrambled” sequence (Lv-shRANDOM) and (3) a vector encoding an efficient shRNA sequence targeting the leptin receptor gene-related protein transcript OB-RGRP [45]. These three vectors were also pseudotyped with the VSV envelope. Cortical glial cells were transduced, as described above, with Lv-shGFAP, Lv-shVIM and the three control vectors, at a single dose of 10 viral particles per seeded cell. We assessed the effects of the combinational silencing of GFAP and vimentin, by also cotransducing glial cells with both the Lv-shGFAP and Lv-shVIM vectors. In these cultures, only glial cells transduced with Lv-shGFAP had significantly lower levels of GFAP (**Fig. 2E**). Glial cells transduced Lv-shVIM or with both Lv-shGFAP and Lv-shVIM vectors had significantly lower levels of vimentin than non transduced cells. By contrast, glial cells transduced with the three control vectors presented levels of GFAP and vimentin similar to those in non transduced cells. Interestingly, glial cells transduced with the sole Lv-shGFAP also presents lower levels of vimentin.

**Figure 3:**
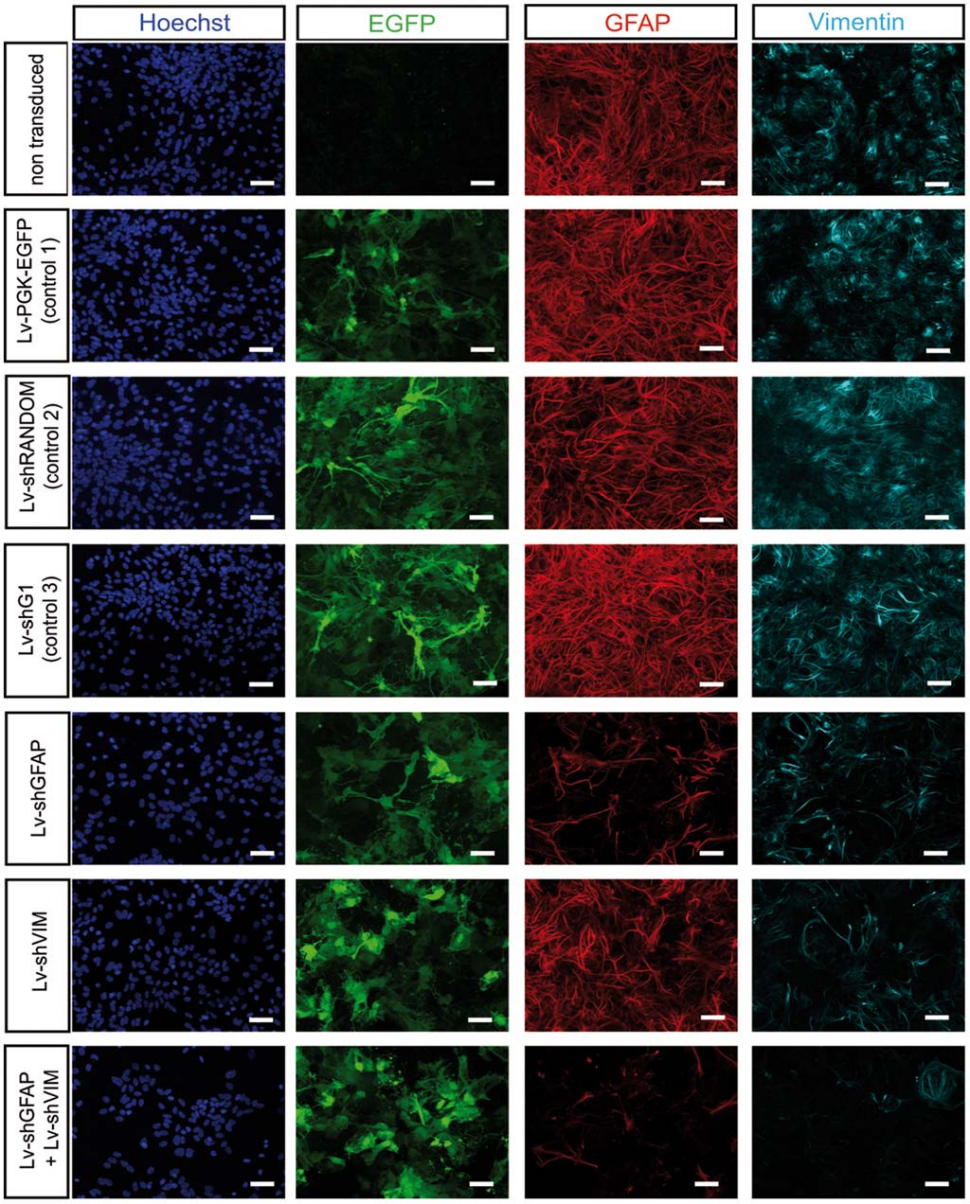
Knockdown by lentiviral-mediated RNAi methods of endogenous GFAP and vimentin levels in primary cultures of medullar astrocytes. GFAP and vimentin levels were decreased in medullar astrocytes transduced with Lv-shGFAP and LvshVIM, respectively. The levels of both proteins were strongly decreased in astrocytes transduced simultaneously with Lv-shGFAP and Lv-shVIM. Astrocytes were cultured from the spinal cords of P2 C57Bl/6 mice. The cells were infected with the Lv-PGK-EGFP, Lv-shRANDOM, Lv-shG1, Lv-shGFAP, Lv-shVIM or with both Lv-shGFAP and Lv-shVIM, at an MOI of 10 viral particles per cell, one week after seeding. Immunostaining for perikaryon detection (Hoechst), EGFP, GFAP and vimentin was performed two weeks after transduction. Scale bar = 50 µm.

In parallel, we detected GFAP and vimentin levels by immunocytochemistry in spinal glial cell cultures after transduction with Lv-shGFAP, Lv-shVIM or the control vectors (**Fig. 3**). Specific immunolabeling of GFAP and vimentin showed that Lv-shGFAP and Lv-shVIM induced a specific and significant decrease in GFAP and vimentin levels, respectively, in these cultures. Moreover, as described above for cortical glial cells, spinal glial cells cotransduced with both Lv-shGFAP and Lv-shVIM also displayed a more pronounced decrease in the levels of both proteins than did cells undergoing single transduction.

Altogether, both western blots of cortical glial cells and immunocytochemical analyses of the spinal cord glial cells showed that Lv-shGFAP and Lv-shVIM efficiently silenced endogenous GFAP and vimentin gene expression.

### GFAP and vimentin knockdown by lentiviral-mediated RNAi decreases glial scarring in primary astrocyte cultures

Astrocytes devoid of GFAP and vimentin present low levels of astroglial reactivity after spinal cord hemisection *in vivo* [32]. Moreover, previous studies have shown that the inhibition of GFAP production by RNA antisense methods decreases astrocyte hypertrophy in a scratch wound model [46]. We evaluated the functional effect of the Lv-shGFAP and Lv-shVIM vectors on the astroglial response *in vitro*, by immunolabeling, to characterize the astrocytic hypertrophy and hyperplasia of scratched primary glial cell cultures transduced with Lv-shGFAP and Lv-shVIM.

**Figure 4:**
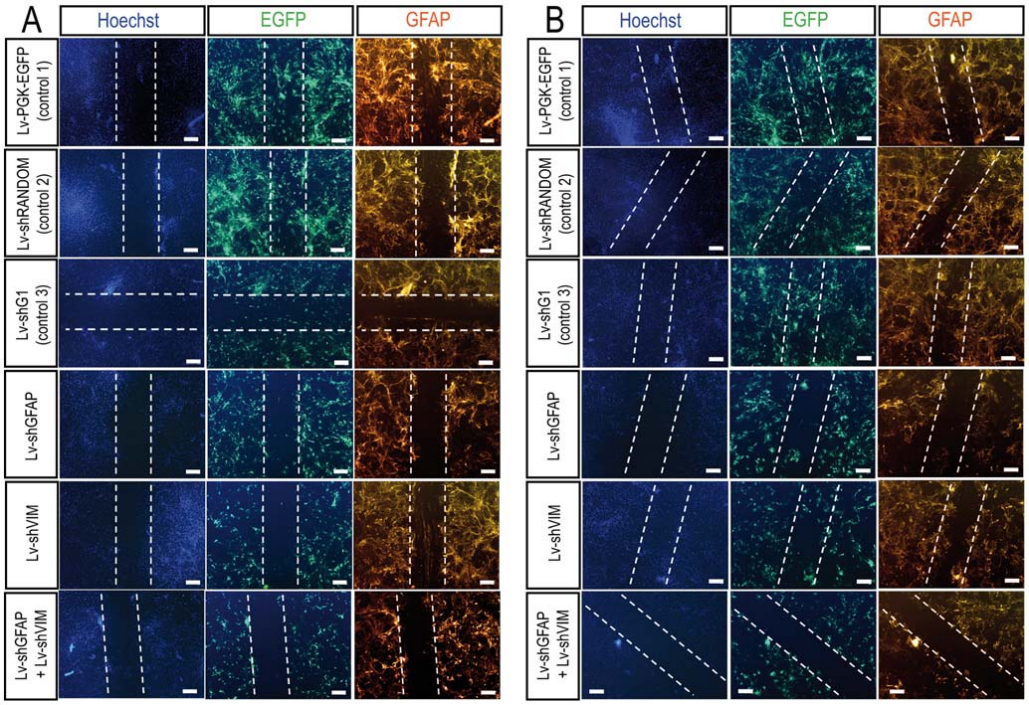
Astrocytic behavior in a scratch wound assay *in vitro* - 1^st^ paradigm - 48 hours and one week after the scratch wound. Cell invasion and GFAP immunostaining are considerably reduced in the scratched area in glial cell cultures transduced with Lv-shGFAP alone or together with Lv-shVIM 48 hours (A) or one week (B) after the scratch wound. Astrocytes were cultured from the spinal cords of P2 C57Bl/6 mice. The cells were infected with Lv-PGK-EGFP, Lv-shRANDOM, Lv-shG1, Lv-shGFAP, Lv-shVIM or with both Lv-shGFAP and Lv-shVIM, at an MOI of 10 viral particles per cell, one week after seeding. The scratch wound was analyzed two weeks after transduction, and immunostaining for perikaryon detection (Hoechst), EGFP, and GFAP was performed 48 hours (A) or one week (B) after the scratch wound. Dashed lines indicate the precise location of the scratch wound. Scale bar = 100 µm.

In a first paradigm, we carried out scratch wound assays on astrocytes previously transduced with 10 particles per cell for each vector. The scratch was performed two weeks after transduction, and the cells were fixed 48 hours, one week or two weeks after scratching. At these time points, the total number of cells invading the scratch area was evaluated by Hoechst staining. Efficiently transduced cells were visualized by EGFP immunolabeling and astrocytic hypertrophy was assessed by determining GFAP immunoreactivity. We first observed invasion of the scratch area by EGFP-positive cells in the three different control conditions 48 hours after scratching (**Fig. 4A** **and** **Fig. S1A**). At this time, the lesioned area was still well outlined by GFAP staining, and slight astrocytic hypertrophy was detected. In glial cell cultures transduced with the Lv-shGFAP vector, total cell invasion of the scratch area was less marked than observed for controls. In particular, fewer EGFP-positive cells were found to be present in the scratch area. GFAP staining also showed levels of glial reactivity to be lower in the vicinity of the lesioned area. Similar observations were obtained for scratched glial cell cultures transduced with both Lv-shGFAP and Lv-shVIM. By contrast, glial cell cultures transduced with Lv-shVIM alone displayed lower levels of cell invasion in the scratched area, but no reduction of GFAP staining.

One week after scratching (**Fig. 4B** **and** **Fig. S1B**) and two weeks after scratching (**Fig. S2A and Fig. S2B**), the decrease in size of the scratched area was much more pronounced in glial cell cultures transduced with the control vectors. Indeed, the lesion was barely detectable two weeks after scratching in these conditions. At both of time points, many large EGFP-positive cells had invaded the scratched area. These cells presented large cytoplasmic processes, characteristic of the astrogliosis observed along the scratch in astrocyte cultures. GFAP immunostaining also revealed strong glial reactivity and astrocytic hypertrophy in the vicinity of the scratched region. By contrast, in cultures transduced with Lv-shGFAP alone or cotransduced with both Lv-shGFAP and Lv-shVIM, astrocytes failed to repair the scratch wound, with fewer cells invading the lesioned area, even two weeks after scratching. Cells transduced with Lv-shGFAP alone or together with Lv-shVIM vector were also smaller than cells transduced with the control vectors, and had smaller cytoplasmic processes. Moreover, GFAP immunostaining revealed astrocytic hypertrophy to be much milder in these cultures. As previously reported, glial cell cultures transduced with Lv-shVIM alone had an intermediate phenotype, one and two weeks after scratching, characterized by lower levels of cellular invasion in the scratch zone and strong residual GFAP immunoreactivity.

In a second paradigm, we assessed the glial response in primary cultures of astrocytes transduced with lentiviral vectors after scratching. Two weeks after seeding, primary glial cell cultures were scratched, and then directly transduced with the various lentiviral vectors. Astrocytic hyperplasia and hypertrophy were analyzed by immunolabeling, as described above. We found that, 48 hours after scratching/transduction the scratch areas and cell morphology were similar in cells transduced with Lv-shGFAP, Lv-shVIM or the control vectors (data not shown).

**Figure 5:**
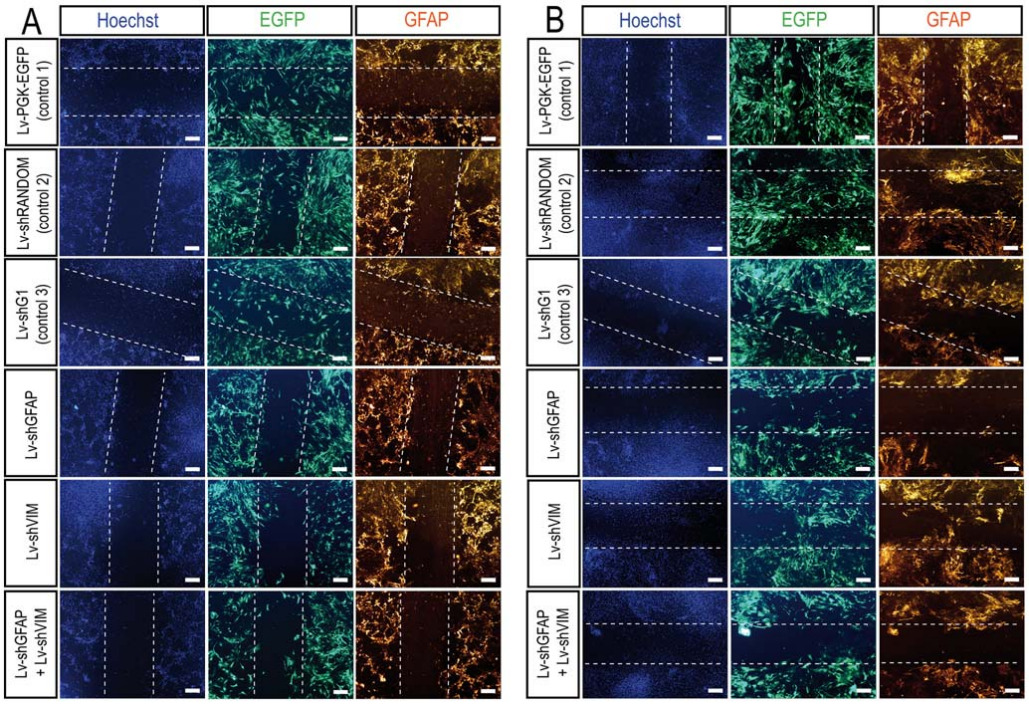
Astrocytic behavior in a scratch wound assay *in vitro* - 2^nd^ paradigm - one week and two weeks after the scratch wound. Cell invasion and GFAP immunostaining are considerably reduced in the scratched area in glial cell cultures transduced with Lv-shGFAP alone or together with Lv-shVIM one week (A) or two weeks (B) after the scratch wound and transduction with the lentiviral vectors. Astrocytes were cultured from the spinal cords of P2 C57Bl/6 mice. The scratch wound was assayed two weeks after cell seeding. The cells were infected with Lv-PGK-EGFP, Lv-shRANDOM, Lv-shG1, Lv-shGFAP, Lv-shVIM or with both Lv-shGFAP and Lv-shVIM, at an MOI of 10 viral particles per cell, directly after the scratch wound. Immunostaining for perikaryon detection (Hoechst), EGFP, and GFAP was performed one week (A) or two weeks (B) after the scratch wound/transduction. Dashed lines indicate the precise location of the scratch wound. Scale bar = 100 µm.

One week after scratching/transduction, scratching areas were clearly detectable in all transduction conditions, as revealed by Hoechst labeling of the nuclei (**Fig. 5A**). Nevertheless, scratched areas in cultures transduced with the control vectors presented a progressive invasion of EGFP-positive cells, whereas those in cultures transduced with Lv-shGFAP and Lv-shVIM vectors did not. Two weeks after scratching/transduction, glial cell cultures transduced with the control vectors presented advanced wound healing, with major colonization by EGFP-positive cells (**Fig. 5B** **and** **Fig. S3**). By contrast, in glial cell cultures transduced with Lv-shGFAP alone or together with Lv-shVIM, the scratched area remained clearly visible, with limited wound healing. In these cultures, GFAP immunoreactivity was also weak and limited to the vicinity of the scratched area. Cultures transduced with Lv-shVIM presented an intermediate phenotype, as previously described, with stronger GFAP immunoreactivity and limited wound healing.

These results demonstrate that Lv-shGFAP, alone or in combination with Lv-shVIM, can significantly decrease astrocyte reactivity and glial scarring *in vitro*. Whether transduction of the astrocytes precedes or is concomitant to the scratching, Lv-shGFAP appears to modulate astroglial reactivity on both hyperplasia and hypertrophy aspects.

### GFAP and vimentin silencing by lentiviral-mediated RNAi increases neuronal survival

We next investigated whether GFAP and/or vimentin silencing by lentiviral-mediated RNA interference also influenced neuronal survival. In a heterotopical coculture model, we analyzed the survival of neurons growing on astrocytes previously transduced with Lv-shGFAP and Lv-shVIM.

**Figure 6:**
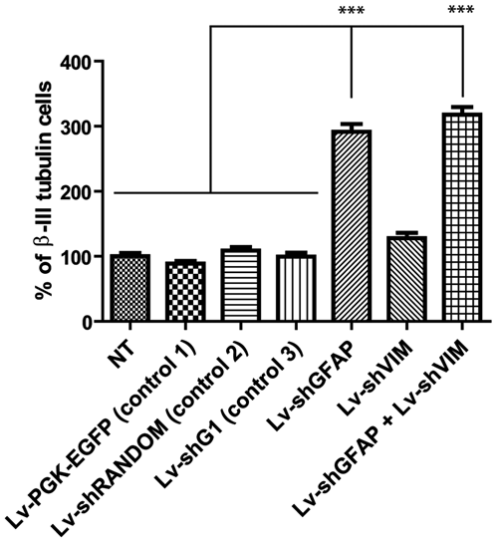
Cortical neuronal survival on cultured medullar astrocytes transduced with the Lv-shGFAP and Lv-shVIM vectors. Quantification of β-III-tubulin-positive cells per square millimeter cocultured on astrocyte monolayers transduced with the various control vectors, Lv-shGFAP, Lv-shVIM, or with both Lv-shGFAP and Lv-shVIM, at an MOI of 10 viral particles per cell. Note the significantly higher survival for neurons growing on astrocytes transduced with Lv-shGFAP alone or with both Lv-shGFAP and Lv-shVIM (***p<0.001, one way ANOVA with Bonferoni *post hoc* test).

We transduced primary glial cell cultures from spinal cord, as described above, with the Lv-shGFAP, Lv-shVIM or control vectors, using the same total amount of vector (MOI = 10) in each case. Two weeks after transduction, we seeded embryonic day-14 wild-type neocortical neurons onto the confluent spinal glial cell cultures. β-III-tubulin-immunoreactive cells were counted after seven days of coculture (**Fig. 6**). We detected no significant difference in neuronal density between cocultures transduced with the control vectors and non transduced cocultures. By contrast, neuronal density appeared to be significantly higher (mean increase of 291.61±13.32% (p<0.001); 2.5 times higher) if neocortical neurons were grown on spinal astrocytes transduced with Lv-shGFAP. In cultures transduced with Lv-shVIM, the proportion of β-III-tubulin-immunoreactive cells was also higher than that for controls, by a mean of 127.72+9.17%, but this increase is significantly different only in comparison with the cells transduced with Lv-PGK-EGFP (p<0.05). Finally, neuronal density was even higher in cocultures cotransduced with both Lv-shGFAP and Lv-shVIM, at 317.67+13.43%, and this difference is extremely significant in comparison with the four controls (p<0.001), but not in comparison with cultures only transduced with Lv-shGFAP, in register with previous findings with KO mice [31]

### Effects of Lv-shGFAP and Lv-shVIM on axonal growth

**Figure 7:**
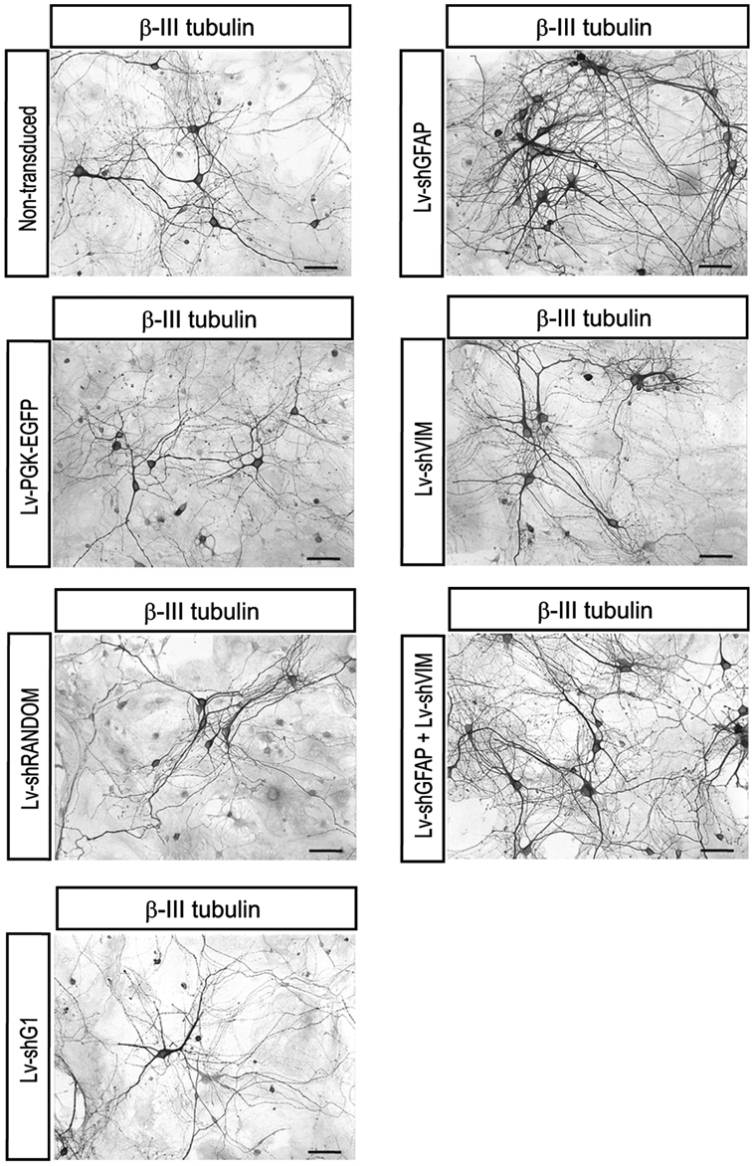
Immunocytochemical characterization of cocultured neurons on astrocyte monolayers transduced with Lv-shGFAP and Lv-shVIM. Neurons were immunocytochemically characterized by β-III-tubulin detection. A high density of β-III-tubulin-positive neurons with a complex network can be observed on astrocytes transduced with the Lv-shGFAP vector or cotransduced with both Lv-shGFAP (E) and Lv-shVIM (G), and, to a lesser extent, on astrocytes transduced with Lv-shVIM alone (F). By contrast, a low density of neurons with few neurite extensions is observed on non transduced astrocytes (A) or on astrocytes transduced with the following control vectors: Lv-PGK-EGFP (B), Lv-shRANDOM (C) and Lv-shG1 (D). Scale bar = 100 µm.

**Figure 8:**
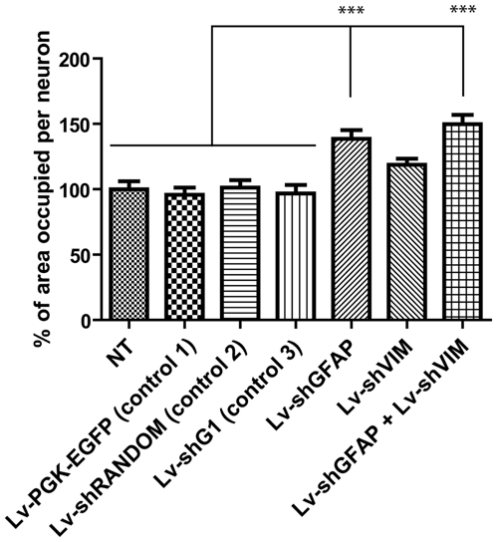
Cortical neurite growth on cultured medullar astrocytes transduced with Lv-shGFAP and Lv-shVIM. Quantification of the percentage of the area occupied per β-III-tubulin-positive cell on astrocyte monolayers transduced with the various control vectors, Lv-shGFAP, Lv-shVIM, or both Lv-shGFAP and Lv-shVIM, at an MOI of 10 viral particles per cell. Greater neurite growth is observed if neurons are cocultured with astrocytes transduced with Lv-shGFAP or with both Lv-shGFAP and Lv-shVIM (***p<0.001, one way ANOVA with Bonferoni *post hoc* test).

We next investigated the effects of Lv-shGFAP and Lv-shVIM on neurite growth, by measuring the percentage of the area occupied per neuron in cocultures. Microscopy after β-III-tubulin immunostaining showed that neurons presented more branching and constituted denser neuropile when they were grown on astrocytes transduced with Lv-shGFAP alone or cotransduced with both Lv-shGFAP and Lv-shVIM vectors, in comparison with the control cocultures (**Fig. 7**). We then quantified the area occupied by the β-III-tubulin immunoreactive neurons growing on astrocytes transduced with the various lentiviral vectors. For each set of conditions, this area was expressed as a percentage, and a value of 100% was attributed to the area occupied by neurons in non transduced cocultures. No significant difference was detected in the areas occupied by neurons grown on non transduced astrocytes and neurons grown on astrocytes transduced with the three control vectors (**Fig. 8**). By contrast, the immunoreactive area occupied per neuron was significantly higher, by a mean of 138±5.2% (p<0.001), if neurons were grown on astrocytes transduced with Lv-shGFAP. In cocultures transduced with Lv-shVIM alone, the area occupied per neuron was also increased but not significantly higher, by 118.8±3.73% in comparison with all the controls. Finally, the area occupied per neuron was even significantly higher, by a mean of 149.79% (p<0.001), in cocultures cotransduced with both Lv-shGFAP and Lv-shVIM. At variance, and as observed with neuronal survival assay, statistical analysis did not reveal a significant difference in the neuritic surface between the cells cotransduced with Lv-shGFAP and Lv-shVIM, and cells only transduced with Lv-shGFAP.

## Discussion

Several therapeutic strategies have been developed in the last few decades to promote axonal regeneration after CNS lesions by modifying the non permissive environment produced either by the glial scar or by myelin debris [47]. Several of these strategies aimed at neutralizing the inhibitory factors produced by the reactive astrocytes, such as chondroitin sulfate proteoglycans [48], [49] and ephrins [50], or at restoring a permissive substrate for axonal growth [51], [52]. In this study, we developed an original gene transfer strategy, making it possible to modulate the intrinsic behavior of reactive astrocytes and to prevent glial scar formation.

Menet *et al*. clearly demonstrated the involvement of GFAP and vimentin upregulation in modulating astrogliosis, *in vitro* and *in vivo* [31], [32], [53]. We developed a molecular tool for targeted, post-transcriptional GFAP and vimentin depletion, to reproduce the physiological effects on astrogliosis and the neuronal response observed in double-mutant animals. RNAi-mediated silencing was efficient, thanks to two factors: i) precise oligonucleotide sequences for triggering the RNAi pathway were identified and ii) an efficient system was used to deliver siRNA to the targeted cells. We first identified a shRNA sequence specific respectively for GFAP and vimentin mRNAs, which significantly reduce the levels of the corresponding proteins fused to the EGFP reporter protein. GFAP and vimentin filaments have been characterized *in vitro* and *in vivo* as polymerized structures with a half-life of eight days [54]. We used integrative lentiviral vectors for the continuous production of shRNAs in astrocytes, to ensure long-term inhibition of both proteins expression. Two lentiviral vectors, Lv-shGFAP and Lv-shVIM, were designed for the expression of the corresponding shRNA sequences and of the EGFP reporter gene as a marker of transduced cells. Western blot analysis showed that these vectors induced a dose-dependent knockdown of endogenous GFAP and vimentin gene expression in cortical glial cell cultures. Fifteen days after transduction, the levels of both proteins were reduced by more than 90% for a dose of 10 viral particles per cell, whereas GFAP and vimentin levels were unaffected in cultures transduced with three different controls. These results were confirmed with immunocytological methods in spinal glial cell cultures. The strong decrease in GFAP and vimentin levels in the transduced glial cells was observed until four weeks after a single transduction, demonstrating the stability of silencing. The lack of change in GFAP and vimentin levels in cultures transduced with Lv-PGK-EGFP (control 1) indicates that the strong decrease in IF proteins level is not due to a toxic effect of the vectors. Moreover, the specificity of RNAi-mediated GFAP and vimentin silencing was confirmed by the lack of effect on GFAP and vimentin levels in cultures transduced with a vector encoding a random sequence (Lv-shRANDOM, control 2) or a vector encoding an shRNA targeting another mRNA (Lv-shG1, control 3).

In light of these results, the efficiency of GFAP and vimentin silencing by lentiviral-mediated RNAi methods may be considered similar to that for other posttranscriptional silencing strategies targeting one or both of these proteins. Two major approaches to reduce GFAP expression have been developed. Eng et al., using a retrovirus-based delivery method and then Lefrançois *et al.*, using Lipofectamine, demonstrated that GFAP production could be inhibited by antisense RNA in primary cultures of astrocytes [46], [55]. Eng *et al*. observed a qualitative decrease in GFAP levels on immunocytochemistry, resulting in a clear decrease in astrocytic hypertrophy [46]. Lefrancois *et al*. showed, by immunoprecipitation, that the efficiency of GFAP inhibition with antisense RNA was about 20 to 30%. Nevertheless, none of these studies reported cellular effects induced by the antisense RNA-mediated inhibition of GFAP beyond 72 hours, and it is therefore difficult to assess the stability of the inhibition achieved with these approaches. Rozovsky *et al.* manipulated GFAP gene expression in rat astrocytes, using a non viral method to deliver RNAi sequences [56]. This resulted in a 30% decrease in the total number of cells producing GFAP. Thus, our approach using vector-based RNAi is currently the most powerful tool for the stable and efficient reduction of endogenous GFAP and vimentin gene expression.

We assessed the cellular effects of the RNAi-mediated silencing of GFAP and vimentin on glial reactivity and neuron-glia interactions. We first analyzed the consequences of the decrease in GFAP and vimentin levels for the scarring process. The ability of Lv-shGFAP and Lv-shVIM to modulate astroglial hypertrophy and hyperplasia was evaluated in a “scratch wound assay” in primary astrocyte cultures. This cellular model is widely used for investigations of the involvement of molecular factors, such as Eph4A [50], tenascin-C [57], Rho GTPase [58] or even GFAP [46] in the glial reactivity. We used this cellular model in two different paradigms. In the first paradigm, we studied the behavior of scratched astrocytes which were previously transduced with Lv-shGFAP and Lv-shVIM. We found that astrocytes transduced with Lv-shGFAP alone or together with Lv-shVIM displayed lower levels of cell invasion of the scratched area and a reduced astrocytic hypertrophy than cultures transduced with control vectors. By contrast, astrocytes transduced with Lv-shVIM alone presented an intermediate phenotype, with a reduction of cell invasion and a persistent glial hypertrophy.

The cellular response of transduced astrocytes was then assessed in a different set of experimental conditions, in which astrocytes were transduced after the scratching assay. This paradigm reproduces more closely the conditions of a therapeutic approach. In these conditions, we found that Lv-shGFAP alone, or together with Lv-shVIM, decreased astrocytic hypertrophy and cellular invasion of the lesioned area. These results confirm and extent those obtained by Eng. *et al* at 72 hours with antisense RNA, for later time points. Indeed, the results obtained in the second paradigm indicate that the RNAi silencing process has the same cellular effects on glial hypertrophy and cell colonization when induced after the “lesion”.

Neuronal survival and neurite growth are two key elements of axonal regeneration after a lesion. We investigated whether decreasing GFAP and vimentin levels by lentiviral-mediated RNAi methods could influence neuron-glia interactions, as described for GFAP knockout and GFAP/Vim double-knockout mice [31]. We showed, in a heterotopic coculture model, that Lv-shGFAP, but not Lv-shVIM, had significant neurotrophic and neuritogenic effects. Considering both neuronal survival and neurite growth, the effects observed with Lv-shGFAP used alone were similar with those observed with the combination of Lv-shGFAP with Lv-shVIM. These results confirm the relationship between astrocytic GFAP inhibition and increases in neuronal plasticity, as previously reported for KO mice.

Quantitative analyses also showed that cotransduction with Lv-shGFAP and Lv-shVIM did not significantly increase neuronal survival or neurite growth in comparison with single transduction with Lv-shGFAP, by contrast with the results obtained with transgenic mice. These observations may be explained by the intrinsic differences between the transgenesis and RNAi-mediated silencing processes. In terms of cellular and molecular dynamics, brisk RNAi-silencing could not generate the progressive compensatory phenomenon observed in GFAP −/− or Vim −/− mice, particularly as the IFs GFAP and vimentin form copolymers. This hypothesis is consistent with the biochemical analysis of endogenous GFAP and vimentin gene expression after RNAi silencing. We found that transduction with Lv-shGFAP alone led to a decrease in vimentin levels. As the RNAi sequence targeting GFAP does not match the sequence of vimentin mRNA, this effect cannot be due to non specific side effects of RNAi. Our results strongly suggest that, in adults, GFAP is the key modulator in the structural assembly of GFAP/Vim polymers. These results are in accordance with the previous studies on transgenic mice [59], [60] which establish that GFAP is the necessary protein, instead of vimentin, for the formation of normal intermediate filaments in astrocytes. Moreover our observations remain consistent, at the functional level, with our previous studies revealing that GFAP is the key modulator of astroglial reactivity [31], [32].

These results indicate that GFAP is a major target to modify astroglial reactivity and consequently neuron-glia interactions for therapeutic purposes. Precisely, monitoring GFAP levels through the use of lentiviral vectors appears as a powerful approach to an efficient reduction of scarring after lesion formation and promotion of both neuronal survival and neuritogenesis.

In conclusion, our *in vitro* findings indicate that Lv-shGFAP, alone or in combination with Lv-shVIM, constitutes a powerful molecular tool for limiting astrocyte reactivity and promoting axonal plasticity. This study provides the necessary proof-of-principle that the manipulation of reactive astrocytes with Lv-shGFAP can be used to stimulate axonal regeneration in the injured CNS. In the perspective of a potential therapeutic use, this strategy is appropriate for an application in animal models of CNS injury. The intrinsic properties of lentiviral vectors make them particularly suitable for gene transfer into the CNS [44]. Sustained transgene expression, even after cell division, is essential for the manipulation of astroglial reactivity, which involves a significant rate of cell multiplication. The possibility of pseudotyping lentiviral vectors to target astrocytes is also a considerable advantage [61]. Thus, the application of Lv-shGFAP and Lv-shVIM in mouse models of spinal cord injury would be of particular relevance for studying *in vivo* the effects of these vectors on glial scar formation and axonal regeneration. Moreover, these tools can be of value for adjunct therapy in several CNS pathological conditions, such as Parkinson disease, in which astrocyte reactivity is an impediment to spontaneous axonal plasticity [62].

## Supporting Information

Figure S1 **Astrocytic behavior in a scratch wound assay *in vitro* - 1st paradigm - two weeks after the scratch wound.** Cell invasion and GFAP immunostaining are considerably reduced in the scratched area in glial cell cultures transduced with Lv-shGFAP alone or together with Lv-shVIM two weeks after the scratch wound. Astrocytes were cultured from the spinal cords of P2 C57Bl/6 mice. The cells were infected with Lv-PGK-EGFP, Lv-shRANDOM, Lv-shG1, Lv-shGFAP, Lv-shVIM or with both Lv-shGFAP and Lv-shVIM, at an MOI of 10 viral particles per cell, one week after seeding. The scratch wound was analyzed two weeks after transduction, and immunostaining for perikaryon detection (Hoechst), EGFP, and GFAP was performed two weeks after the scratch wound. Dashed lines indicate the precise location of the scratch wound. Scale bar = 100 µm.
(9.51 MB TIF)

Figure S2 **Astrocytic behavior in a scratch wound assay *in vitro* - 1st paradigm.** Cell invasion and GFAP immunostaining are considerably reduced in the scratched area in glial cell cultures transduced with Lv-shGFAP alone or together with Lv-shVIM 48 hours (**A**), one week (**B**) or two weeks (**C**) after the scratch wound. Astrocytes were cultured from the spinal cords of P2 C57Bl/6 mice. The cells were infected with Lv-PGK-EGFP, Lv-shRANDOM, Lv-shG1, Lv-shGFAP, Lv-shVIM or with both Lv-shGFAP and Lv-shVIM, at an MOI of 10 viral particles per cell, one week after seeding. The scratch wound was analyzed two weeks after transduction, and immunostaining for perikaryon detection (Hoechst), EGFP, and GFAP was performed 48 hours (**A**), one week (**B**) or two weeks (**C**) after the scratch wound. Dashed lines indicate the precise location of the scratch wound. Scale bar = 50 µm.
(6.00 MB TIF)

Figure S3 **Astrocytic behavior in a scratch wound assay *in vitro* - 2nd paradigm - two weeks after the scratch wound.** Cell invasion and GFAP immunostaining are considerably reduced in the scratched area in glial cell cultures transduced with Lv-shGFAP alone or together with Lv-shVIM two weeks after the scratch wound and transduction with the lentiviral vectors. Astrocytes were cultured from the spinal cords of P2 C57Bl/6 mice. The scratch wound was assayed two weeks after cell seeding. The cells were infected with Lv-PGK-EGFP, Lv-shRANDOM, Lv-shG1, Lv-shGFAP, Lv-shVIM or with both Lv-shGFAP and Lv-shVIM, at an MOI of 10 viral particles per cell, directly after the scratch wound. Immunostaining for perikaryon detection (Hoechst), EGFP, and GFAP was performed two weeks after the scratch wound/transduction. Dashed lines indicate the precise location of the scratch wound. Scale bar = 50 µm.
(8.76 MB TIF)
