# Supplementary figures and images for: A Novel and Efficient Gene Transfer Strategy Reduces Glial Reactivity and Improves Neuronal Survival and Axonal Growth In Vitro
Source: PLoS One. 2009 Jul 14;4(7):e6227. doi: 10.1371/journal.pone.0006227 (PMC2705675; doi:10.1371/journal.pone.0006227)

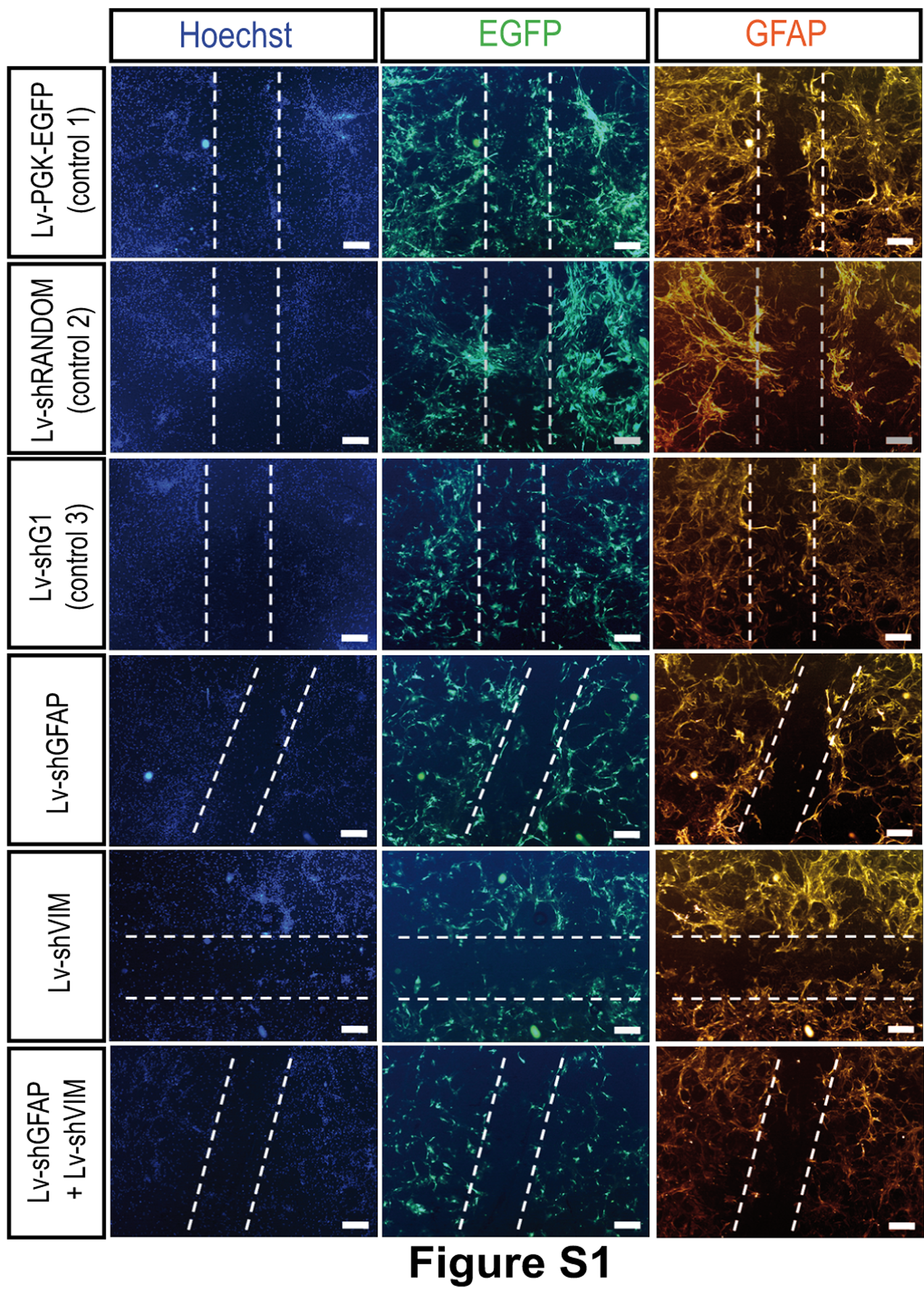

Supplement: Figure S1 — Astrocytic behavior in a scratch wound assay in vitro - 1st paradigm - two weeks after the scratch wound. Cell invasion and GFAP immunostaining are considerably reduced in the scratched area in glial cell cultures transduced with Lv-shGFAP alone or together with Lv-shVIM two weeks after the scratch wound. Astrocytes were cultured from the spinal cords of P2 C57Bl/6 mice. The cells were infected with Lv-PGK-EGFP, Lv-shRANDOM, Lv-shG1, Lv-shGFAP, Lv-shVIM or with both Lv-shGFAP and Lv-shVIM, at an MOI of 10 viral particles per cell, one week after seeding. The scratch wound was analyzed two weeks after transduction, and immunostaining for perikaryon detection (Hoechst), EGFP, and GFAP was performed two weeks after the scratch wound. Dashed lines indicate the precise location of the scratch wound. Scale bar = 100 µm. (9.51 MB TIF) [file pone.0006227.s001.tif]

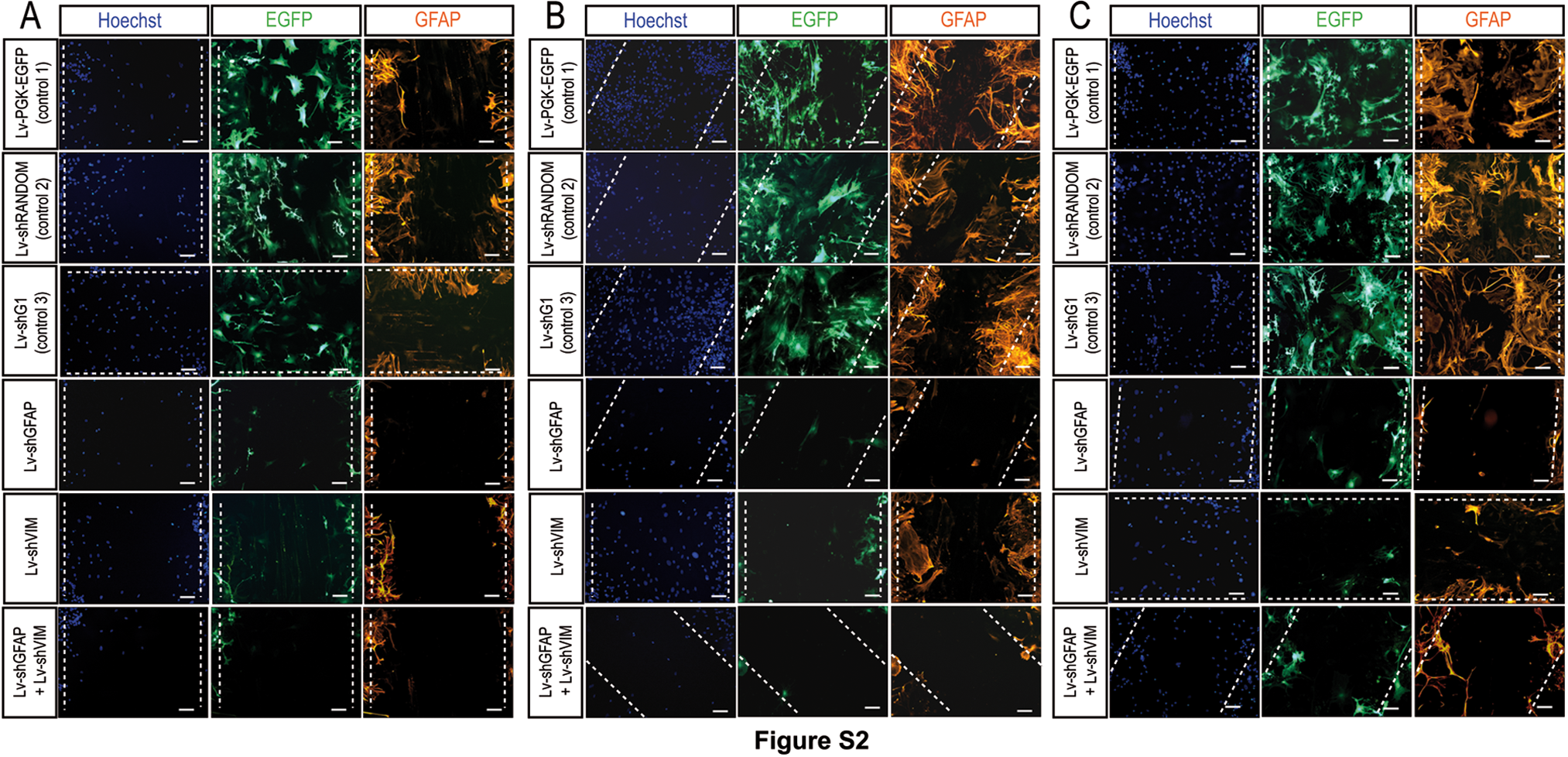

Supplement: Figure S2 — Astrocytic behavior in a scratch wound assay in vitro - 1st paradigm. Cell invasion and GFAP immunostaining are considerably reduced in the scratched area in glial cell cultures transduced with Lv-shGFAP alone or together with Lv-shVIM 48 hours (A), one week (B) or two weeks (C) after the scratch wound. Astrocytes were cultured from the spinal cords of P2 C57Bl/6 mice. The cells were infected with Lv-PGK-EGFP, Lv-shRANDOM, Lv-shG1, Lv-shGFAP, Lv-shVIM or with both Lv-shGFAP and Lv-shVIM, at an MOI of 10 viral particles per cell, one week after seeding. The scratch wound was analyzed two weeks after transduction, and immunostaining for perikaryon detection (Hoechst), EGFP, and GFAP was performed 48 hours (A), one week (B) or two weeks (C) after the scratch wound. Dashed lines indicate the precise location of the scratch wound. Scale bar = 50 µm. (6.00 MB TIF) [file pone.0006227.s002.tif]

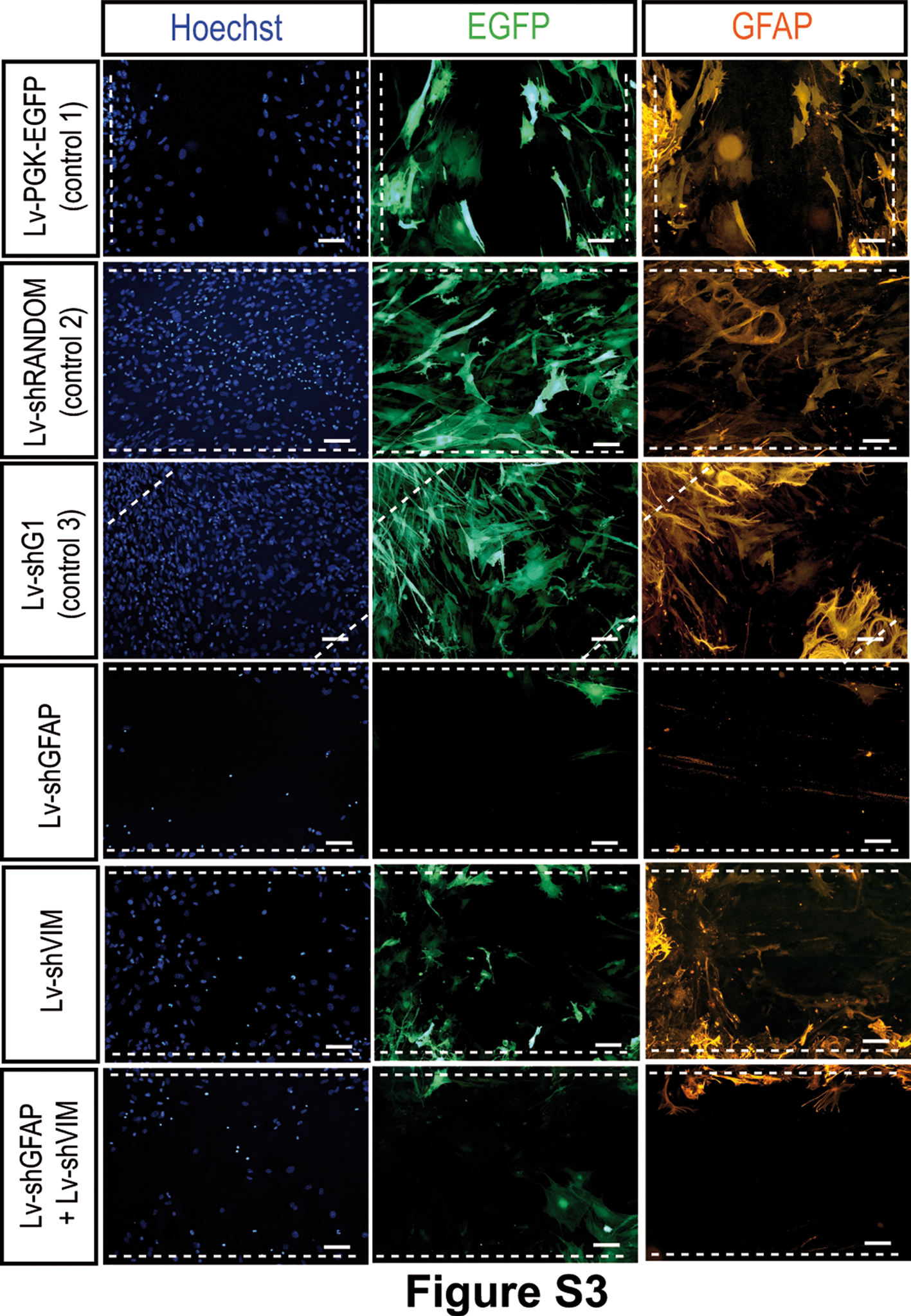

Supplement: Figure S3 — Astrocytic behavior in a scratch wound assay in vitro - 2nd paradigm - two weeks after the scratch wound. Cell invasion and GFAP immunostaining are considerably reduced in the scratched area in glial cell cultures transduced with Lv-shGFAP alone or together with Lv-shVIM two weeks after the scratch wound and transduction with the lentiviral vectors. Astrocytes were cultured from the spinal cords of P2 C57Bl/6 mice. The scratch wound was assayed two weeks after cell seeding. The cells were infected with Lv-PGK-EGFP, Lv-shRANDOM, Lv-shG1, Lv-shGFAP, Lv-shVIM or with both Lv-shGFAP and Lv-shVIM, at an MOI of 10 viral particles per cell, directly after the scratch wound. Immunostaining for perikaryon detection (Hoechst), EGFP, and GFAP was performed two weeks after the scratch wound/transduction. Dashed lines indicate the precise location of the scratch wound. Scale bar = 50 µm. (8.76 MB TIF) [file pone.0006227.s003.tif]
